# Analysis of the In Vitro Toxicity of Nanocelluloses in Human Lung Cells as Compared to Multi-Walled Carbon Nanotubes

**DOI:** 10.3390/nano12091432

**Published:** 2022-04-22

**Authors:** Fátima Pinto, Ana Filipa Lourenço, Jorge F. S. Pedrosa, Lídia Gonçalves, Célia Ventura, Nádia Vital, Ana Bettencourt, Susete N. Fernandes, Rafaela R. da Rosa, Maria Helena Godinho, Henriqueta Louro, Paulo J. T. Ferreira, Maria João Silva

**Affiliations:** 1 National Institute of Health Doutor Ricardo Jorge, Department of Human Genetics, 1649-016 Lisbon, Portugal; fatima.pinto@insa.min-saude.pt (F.P.); celia.ventura@insa.min-saude.pt (C.V.); nadia.vital@insa.min-saude.pt (N.V.); henriqueta.louro@insa.min-saude.pt (H.L.); 2 ToxOmics—Centre for Toxicogenomics and Human Health, NOVA Medical School, Universidade NOVA de Lisboa, 1169-056 Lisbon, Portugal; 3 RAIZ—Forest and Paper Research Institute, 3800-783 Eixo, Portugal; ana.filipa@thenavigatorcompany.com; 4 CIEPQPF, Department of Chemical Engineering, University of Coimbra, Pólo II, 3030-790 Coimbra, Portugal; jorge_fsp@live.com.pt (J.F.S.P.); paulo@eq.uc.pt (P.J.T.F.); 5 Research Institute for Medicines (iMed.ULisboa), Faculty of Pharmacy, Universidade de Lisboa, 1649-003 Lisbon, Portugal; lgoncalves@ff.ulisboa.pt (L.G.); asimao@ff.ulisboa.pt (A.B.); 6 NOVA Medical School, Universidade NOVA de Lisboa, 1169-056 Lisboa, Portugal; 7 CENIMAT/I3N, Department of Materials Science, NOVA School of Science and Technology (FCT NOVA), NOVA University Lisbon, Campus da Caparica, 2825-149 Caparica, Portugal; sm.fernandes@fct.unl.pt (S.N.F.); rr.rosa@fct.unl.pt (R.R.d.R.); mhg@fct.unl.pt (M.H.G.)

**Keywords:** micro/nanocelluloses, in vitro cytotoxicity, genotoxicity, micronucleus assay, cell uptake, reactive oxygen species

## Abstract

Cellulose micro/nanomaterials (CMNM), comprising cellulose microfibrils (CMF), nanofibrils (CNF), and nanocrystals (CNC), are being recognized as promising bio-nanomaterials due to their natural and renewable source, attractive properties, and potential for applications with industrial and economical value. Thus, it is crucial to investigate their potential toxicity before starting their production at a larger scale. The present study aimed at evaluating the cell internalization and in vitro cytotoxicity and genotoxicity of CMNM as compared to two multi-walled carbon nanotubes (MWCNT), NM-401 and NM-402, in A549 cells. The exposure to all studied NM, with the exception of CNC, resulted in evident cellular uptake, as analyzed by transmission electron microscopy. However, none of the CMNM induced cytotoxic effects, in contrast to the cytotoxicity observed for the MWCNT. Furthermore, no genotoxicity was observed for CNF, CNC, and NM-402 (cytokinesis-block micronucleus assay), while CMF and NM-401 were able to significantly raise micronucleus frequency. Only NM-402 was able to induce ROS formation, although it did not induce micronuclei. Thus, it is unlikely that the observed CMF and NM-401 genotoxicity is mediated by oxidative DNA damage. More studies targeting other genotoxicity endpoints and cellular and molecular events are underway to allow for a more comprehensive safety assessment of these nanocelluloses.

## 1. Introduction

In recent years, there has been growing interest in the development of environmentally friendly materials and processes, which has motivated the use of sustainable biopolymers such as cellulose. This abundant bio-based polymer is composed of β-1,4-linked anhydro-D-glucose units and can be isolated from a wide variety of natural sources such as wood (hardwood and softwood), seed fibers (cotton, coir, etc.), grasses, marine animals, and algae, or are generated by fungi, invertebrates, and bacteria [1,2,3]. Industrially, cellulose derives primarily from woody plants in the form of cellulose fibers, where it exists in combination with other constituents (mainly hemicellulose and lignin), arranged as an organized structure of fibrillary elements [2]. Cellulose micro/nanomaterials (CMNM), obtained from the deconstruction of cellulosic fibers into their hierarchical sub-structures, have been extensively studied for their application in diverse industrial fields that were once thought to be impossible for conventional cellulosic materials [4,5,6]. Depending on the source and production method, CMNM present unique physicochemical properties such as high specific area, high tensile strength and stiffness, gas barrier properties, optical properties, biodegradability, and biocompatibility, which make them a promising alternative material for synthetic products [7,8,9]. 

Two main types of CMNM, namely cellulose micro/nanofibrils (CMF/CNF) and cellulose nanocrystals (CNC), have received much attention for their use in scaffolds for bone tissue regeneration, drug delivery, substrates for human stem cell cultures, wound dressings, food packaging, cosmetics, the production of lightweight and durable films, among others [10,11,12,13,14,15]. CMF/CNF are described as semi-crystalline cellulosic fibril aggregates with high aspect ratios, diameters inferior to 100 nm (in the case of CNF), and lengths in the micrometer scale [16,17,18]. CMF/CNF are generally produced by subjecting the cellulose fibers to an intensive mechanical treatment, for example, high-pressure homogenization (HPH) and/or grinding. The mechanical treatment is typically preceded by a chemical or an enzymatic pre-treatment to reduce energy consumption [4,19]. CNC, also referred to as cellulose nanocrystals, cellulose whiskers, or nanocrystalline cellulose are rod-like or needle-like colloidal particles that derive from the crystalline regions within elementary nanofibrils of cellulose and are isolated from the cellulose amorphous domains of these nanofibrils [4,20,21,22]. They present an elongated, rod-like shape, with a length ranging from 100 nm to several μm and a width of 3–30 nm, a high degree of crystallinity (50–90%), and a smaller aspect ratio and limited flexibility as compared to CNFs, which are dependent on the extraction process and cellulose source [2,3,23]. CNC are commonly prepared through a strong acid hydrolysis under strictly controlled conditions of temperature, vigorous agitation, and time; in these conditions, the amorphous regions of cellulose are preferentially hydrolyzed, while the crystalline regions remain intact because of their inherent structural stability [23,24]. 

The growing number of CMNM applications in multiple products at an industrial scale, including in the biomedical area, should be preceded by the assessment of their safety to human health [25,26]. Inhalation is considered to be the main route of exposure throughout the lifecycle of CMNM, representing a potential hazard both to workers and to consumers [27]. Considering the natural basis of cellulose, CMNM are frequently described as nontoxic. However, the biopersistence of these nanomaterials (NM) in the lungs and their high aspect ratio [28,29,30,31] are features similar to those of other fibrous NM (e.g., multi-walled carbon nanotubes (MWCNT) or asbestos fibers) with related adverse effects on human health, such as the development of bronchogenic carcinoma, malignant mesothelioma, and interstitial pulmonary fibrosis (asbestosis) [16,32]. Additionally, some properties of CMNM such as, nanofiber size, morphology, degree of crystallinity, surface chemistry, aggregation state, among other unknown characteristics, may determine their biological behavior in comparison with macro-scale materials and dictate their toxicity [23,33]. As a result, before scaling up their production and commercialization, it is extremely important to address the potential toxicological properties of CMNM, particularly their cytotoxicity and genotoxicity, which are closely associated with carcinogenicity [16,34]. Despite the increasing interest in this subject and the number of recent reports in the literature [13,34], there is still limited information and contradictory findings about the toxicological aspects of CMNM. The inconsistency of the results may be related to different variables such as the cellulose source, the type or non-use of pre-treatment, and the mechanical fibrillation procedure, which can originate CMNM with distinct properties that affect their toxicity [35]. Considering a few literature reports, CNC exposures are associated with cytotoxicity and immunotoxicity in vitro and in vivo [26,36,37,38,39,40]. However, there are only a few studies evaluating the genotoxicity of CNC, which report discrepant results [13,41]. Kisin et al., 2020 [13] showed that short-term exposure of human pulmonary epithelial cells (BEAS-2B) to CNC derived from wood induced DNA damage and intracellular ROS increase, while long-term exposure resulted in neoplastic-like transformation. However, Catalán et al., 2015 [41] reported negative results for genotoxicity assessed through the exposure of BEAS-2B cells to cotton CNC (average length, 135 ± 5 nm; width, 7.3 ± 0.2 nm) with a concentration range of 2.5–100 μg/mL, evaluated by micronucleus assay. Concerning the toxic effects of CNF, a small number of literature reports generally indicate no relevant cytotoxic, genotoxic, or immunotoxic effects [36,42,43,44,45,46,47,48]. Genotoxic effects of CNF obtained from brown and curauá cotton were observed in human lymphocytes and 3T3 cell mouse fibroblasts [49]. Additionally, Ventura et al. 2018 [16], observed an increase in the frequency of micronuclei in A549 cells co-cultured with THP-1 macrophages and exposed to 1.5 and 3 μg/cm^2^ of CNFs obtained from bleached *Eucalyptus globulus* kraft pulp by TEMPO (2,2,6,6-tetramethylpiperidine 1-oxyl)-mediated oxidation (width, 25.9 nm). No genotoxic effects of CNF were observed through the alkaline comet assay and cytokinesis-block micronucleus assay in BEAS-2B cells, but an increase in intracellular reactive oxygen species (ROS) formation was observed [34]. It is thus necessary to investigate the potential respiratory effects of CMNM in vitro. 

The purpose of the present study was to evaluate the potential toxic effects of CMNM derived from bleached *Eucalyptus globulus* kraft pulp in human lung epithelial cells (A549) and to compare them with two MWCNT (NM-401 and NM-402, representative test materials (RTM) from the JRC Repository), in view of their structural resemblance in terms of high aspect ratio and high stiffness. RTM are assumed to be representative of a large fraction of NM on the market, consisting of a (random) sample from one industrial production batch produced within industrial specifications and later sub-sampled into vials under reproducible (GLP) conditions [50]. These MWCNT are identified as being genotoxic in A549 cells through the cytokinesis-block micronucleus assay [51]; more specifically, the NM-401, which is more rigid, showed significant uptake by cells and induced significant concentration-dependent intracellular ROS, HPRT mutations in Chinese hamster lung (V79) fibroblasts [52], and a pro-inflammatory response in both A549 and THP-1 cells [53]. The human lung adenocarcinoma epithelial cell line (A549) used in this study mimics the function of Type II pneumocytes, retaining the endocytic ability of the pulmonary epithelium and the localization of cytochrome P450 systems [51,54]; it is commonly used as a model for studying pulmonary toxicity [55]. 

This study focused on three micro/nanocellulose samples: CNF produced by catalytic oxidation with TEMPO radical (CNF-TEMPO); CMF produced by enzymatic hydrolysis (CMF-ENZ); CNC produced by acid hydrolysis. More specifically, we assessed the ability of these materials—produced from the same cellulose source but differing in production method, and, consequently in physicochemical properties—to induce cytotoxic effects, the formation of intracellular ROS, and chromosomal damage.

## 2. Materials and Methods

### 2.1. Synthesis and Characterization of Cellulose Micro/Nanofibrils and Nanocrystals 

CNF, CMF, and CNC were obtained from industrially bleached *Eucalyptus globulus* kraft pulp (BEKP). To produce CMF and CNF, and prior to any pre-treatment, 30 g of cellulose fibers were disintegrated and refined at 4000 rev. in a PFI beater. After that, to produce the TEMPO-oxidized CNF, the refined fibers were dispersed in distilled water containing the radical TEMPO (0.016 g/g of fibers) and sodium bromide (NaBr, 0.1 g/g of fibers), according to a methodology described elsewhere [56,57]. The previous mixture was stirred for 15 min at room temperature in order to assure a good dispersion. A NaClO solution (9% active chlorine) was slowly added to the fiber suspension (5 mM/g of fibers), maintaining a pH of 10 by adding drops of NaOH 0.1 M until a stable pH was reached (approximately 2 h). The resultant fibers were then filtered and washed with distilled water until the conductivity of the filtrate reached low values (20 μS/cm). Finally, the TEMPO pre-treated fibers, designated as CNF-TEMPO, were mechanically disintegrated in a high-pressure homogenizer (GEA Niro Soavi, model Panther NS3006 L) with 2 passages, the first one at 500 bar and the second at 1000 bar, in order to reduce their size to the nanoscale. The result was an aqueous gel-like suspension of 0.89 wt % solid content. 

The CMF were prepared according to a methodology described by Lourenço et al. [58]. Briefly, after the first mechanical treatment in a PFI beater, the fibers were suspended in water, and 0.05 M citrate buffer was added until pH 5 was reached. An enzymatic cocktail (10% endocellulase, 10% exocellulase, and 5% hemicellulase) was added (300 g/ton of pulp) to the fiber suspension and kept at 50 °C with constant mechanical stirring for 2 h. The temperature was raised to 80 °C for 15 min to sop the hydrolysis reaction and was then cooled to room temperature. After that, the hydrolyzed cellulose, designated as CMF-ENZ, was thoroughly washed with demineralized water and subjected to a mechanical disintegration step in a high-pressure homogenizer (2 passages, the first one at 500 bar and the second at 1000 bar). The consistency of the final suspension was 0.93 wt %.

The CNC were obtained from BEKP, with diluted sulfuric acid (62 wt %, from 95–97%, Sigma-Aldrich p.a, St. Louis, MO, USA) and an acid solution/solid ratio of 8:1, adapted from an acid hydrolysis method described elsewhere [59,60]. The acid hydrolysis was carried out at 55 °C under mechanical stirring and for 75 min. Next, the mixture was quenched with ultrapure water (10 times the reactional volume). Subsequently, CNC were released from the reaction mixture by several centrifugation cycles, which led to CNC in the supernatant with a pH ranging from 1.3 to 2.5. Further purification was achieved by dialysis against ultrapure water, where the suspended CNC inside a Spectra/Por^®^ 4 Dialysis Tubing (from Spectrum^®^, with an average molar mass cut-off of 12–14 kDa, 45 mm flat width) were purified until a constant pH was reached. To obtain dry CNC in their acid form (pH = 3.3 in suspension), a freeze-dryer was used (−45 °C, at 0.3 mbar, VaCO 2, Zirbus). The CHS (Carbon/Sulfur/Hydrogen) elemental determination was obtained using the Thermo Finnigan-CE Instruments Flash EA 1112 CHNS series analyzer. The measurements were performed in duplicate and are presented as mean values.

CMF and CNF were characterized according to their fibrillation yield, carboxyl content (C_COOH_), degree of polymerization (DP), and intrinsic viscosity ([η]). The fibrillation yield was calculated as the percentage (*w*/*w*) of supernatant material after submitting 0.2 wt % of nanocellulose dispersions to centrifugation at 9000 rpm for 30 min in a Hettich Universal 32 [61]. The results were determined in duplicate. The C_COOH_ content was determined by conductometric titration, according to Lourenço et al. (2017) [57]. Briefly, the pH of the nanocellulose dispersion (0.1 g dry weight) was set to 3.0, and then a 0.01 M NaOH solution was added until pH 11 was reached. The C_COOH_ content was determined in triplicate from the conductivity curve. The intrinsic viscosity, which was necessary to be able to calculate the degree of polymerization (DP) through the Mark–Houwink equation with the parameters reported by Henriksson et al. (2008) [62], was determined by means of the cupri-ethylenediamine methodology (ISO standard 5351:2010).

**Figure 1:**
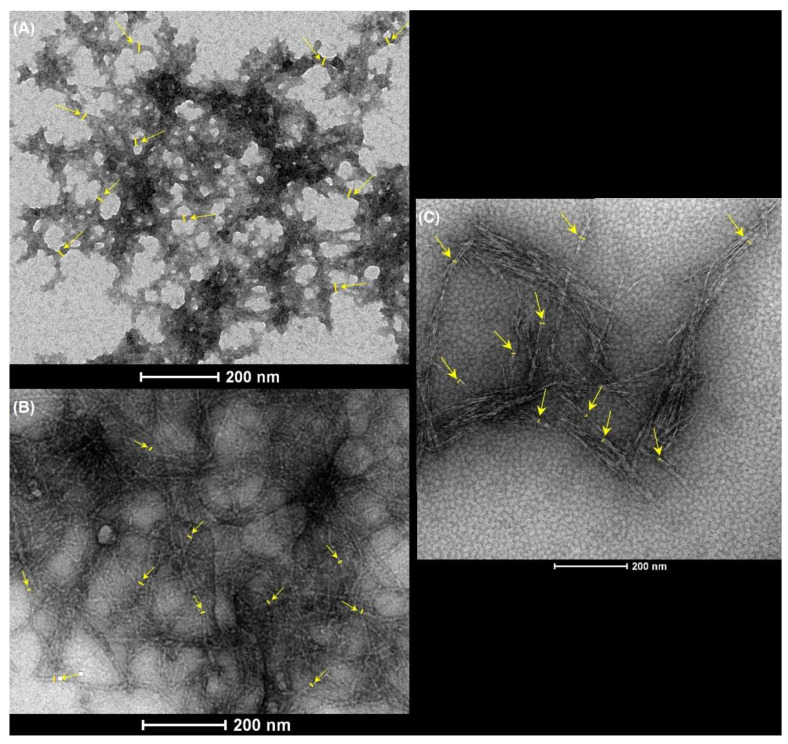
TEM images exemplifying the measurement of the fibril diameter (yellow lines) using the software ImageJ: (**A**) CMF-ENZ, (**B**) CNF-TEMPO, and (**C**) CNC.

The morphology, the hydrodynamic diameter (z-Average), and the surface charge of the three nanocellulose samples were analyzed and diluted in phosphate buffered saline (PBS) and in a complete RPMI 1640 (Gibco, Waltham, MA, USA) culture medium. The morphology and estimated diameter of the cellulose nanofibrils and nanocrystals were investigated by Transmission Electron Microscope (TEM) imaging with the use of the negative staining technique, as follows: copper TEM grids with a formvar carbon support film were placed in a 10 μL drop of nanocellulose suspension and left for 5 min. Then, the sample grids were washed in 10 drops of water and were placed in one drop of 2% uranyl acetate for 5 min. After each step, excess moisture was removed along the periphery of the grid with Whatman 1 filter paper. The grids were examined at 1050 k, 4200 k, and 1100 k for an overview and at 43,000 k, 60,000 k, and 87,000 k for high magnifications using a Tecnai G2 Spirit BioTWIN TEM from FEI equipped with two cameras: the Olympus-SIS Veleta CCD Camera, which is optimal for rapid screening of the sample, and the Eagle 4K HS camera, used for high-resolution data acquisition of sensitive samples. The fibril diameter was estimated through the average of 10 measurements in one image, analyzed using the public domain software Image J, as exemplified in Figure 1. The zeta potential, which reflects the electric charge on the particle surface and indicates the physical stability of colloidal systems, was quantified using a Malvern Zetasizer Nano ZS (Malvern Panalytical Inc., Westborough, MA, USA) by measuring the electrophoretic mobility of the nanocellulose in an electric field, using the Helmholtz–Smoluchowsky equation. Before measurements, 1 mL of nanocellulose diluted in PBS or in culture medium was transferred and placed in a folded capillary cell (DTS1060), to which an alternating voltage of ±150 mV was applied, using a dispersant (water) dielectric constant of 78.5. All measurements were performed at 25 °C, in triplicate, and the mean ± SD value was reported. 

### 2.2. Preparation of the Nanocelluloses and MWCNT Exposure Suspensions

The stock suspensions of nanocellulose at 1.5 mg/mL were prepared by dispersing the CNF-TEMPO and CMF-ENZ gels and the CNC powder in PBS with magnetic agitation for 30 min. The suspensions were then diluted in complete RPMI 1640 cell culture medium in order to prepare the chosen concentrations before exposure to the cells. Bright field microscopy images of the stock dispersion of each CMNM showed dispersed fibrils (CMF-ENZ and CNF-TEMPO) or rods (CNC) and agglomerates/aggregates. 

Two MWCNT were kindly provided by the JRC Repository (NM-401 and NM-402) for use as references. Their physicochemical characterization is summarized in the study by Rasmussen et al. (2014) [50] and in Table 1, showing that they mainly differ in diameter, length, and flexibility. The stock suspensions of these two NM at 2.56 mg/mL were prepared according to the NANOGENOTOX dispersion protocol [63], as follows: the NM were dispersed in sterile-filtered 0.05 % wt BSA-water with 0.5% absolute ethanol (96%) and then dispersed for 15 min using an ultrasonic homogenizer 400-Watt Branson Sonifier S-450D (Branson Ultrasonics Corp., Danbury, CT, USA), as previously described [52]. The physicochemical properties of the MWCNTs as well as their hydrodynamic size distributions and zeta potential in the stock dispersions and in the culture medium have already been determined by dynamic light scattering (DLS) and published elsewhere [64]. 

### 2.3. Cell Culture and Exposure of Nanofibers

The A549 cell line was obtained from the American Type Culture Collection (ATCC, Manassas, VA, USA, CCL-185). A549 cells were cultured in complete culture medium (CM) consisting of RPMI 1640 medium (Thermo Fisher Scientific, Waltham, MA, USA) supplemented with 10% heat-inactivated fetal bovine serum (FBSi, Thermo Fisher Scientific), 1% penicillin/streptomycin (1.000 U/mL penicillin and 10 mg/mL streptomycin (Thermo Fisher Scientific), and 1% fungizone (0.25 mg/mL, Thermo Fisher Scientific) at 37 °C in an atmosphere of 5% CO_2_. Log-phase A549 cells were inoculated into a 96-well plate (MTT, PI assay, and ROS) and into a 6-well plate (clonogenic and micronucleus assays) and cultured as a monolayer for 24 h before the exposure. Semi-confluent cell cultures were exposed to the CMNM or MWCNT samples and kept at 37 °C in 5% CO_2_ during the exposure time.

### 2.4. Cytotoxicity Assessment

#### 2.4.1. MTT Assay

The A549 cells were plated at 0.5 × 10^4^ cells/well in a 96-well plate and allowed to adhere for 24 h at 37 °C in a humidified atmosphere of 5% CO_2_. The cells were then exposed to 1.5, 3, 6, 12.5, 25, and 50 μg/cm^2^ of each nanofiber in a culture medium for 24 h. SDS (0.1 mg/mL, Sigma) was used as a positive control, with the cells exposed for 1 h. After that, the culture medium was removed, and the cells were washed twice with PBS and then replaced with a basal medium containing 10% of the MTT solution (5 mg/mL, Calbiochem, Darmstadt, Germany). The cells were incubated for another 4 h, after which dimethyl sulfoxide (DMSO, Sigma) was added for 20 min to dissolve the formazan crystals for spectrophotometric quantification. The absorbance at 570 nm was recorded against a reference filter set at 690 nm using a Multiscan Ascent spectrophotometer (Labsystems, Helsinki, Finland). The relative cell survival of exposed cultures was expressed as the ratio between the absorbance of exposed and unexposed cells, presuming that the absorbance of the unexposed cells represents 100% cell survival. Three independent assays were performed, with a minimum of three replicates per concentration. The potential interference of the MWCNT with the MTT assay was checked by comparing the absorbance values read at the end of the assay and the absorbance read after the centrifuge and transference of the supernatant to new plates.

#### 2.4.2. Clonogenic Assay

Exponentially growing A549 cells were plated in 6-well microplates at a density of 200 cells/well. After approximately 14 h, the cells were exposed to 1.5, 3, 6, 12.5, 25, and 50 μg/cm^2^ of each nanofiber in the culture medium. For each experiment, negative (non-treated cells) and positive (0.004 μg/mL mitomycin C, Sigma) controls were included. To ensure that mostly single cells are in the culture at the time of exposure, the period between cell plating and exposure should not exceed the population doubling time, which has been determined to be about 22 h for A549 cells (ATCC, CCL-185). The cells were incubated at 37 °C in a humidified atmosphere of 5% CO_2_ and left for 8 days, which is the time necessary to form colonies, a colony being defined as having at least 50 cells. 

At the end of the exposure period, the cell culture medium was removed, and the cells were washed twice with PBS and were fixed using absolute cold methanol (Sigma). Finally, before the colonies were counted, the cells were stained using 10% Giemsa (Merck, Darmstadt, Germany) that was prepared in Gurr’s phosphate buffer. The plating efficiency (PE) was determined using the following equation:

(1) $$\mathrm{PE}=\frac{No.\;of\;colonies\;formed\;Unexposed}{No.\;of\;cells\;seeded}\times 100$$

The surviving fraction (SF) for each nanofiber concentration was calculated as follows:

(2) $$\mathrm{SF}=\frac{No.\;of\;colonies\;formed\;Exposed}{No.\;of\;cells\;seeded\times PE/100}$$

The cytotoxicity was determined as the decrease of the SF when compared to the negative control, based on the results from three independent experiments with six replicates for each concentration.

#### 2.4.3. Propidium Iodide (PI) Membrane Integrity Assay

Cell viability was assessed after a 24 h exposure period to different concentrations (1.5, 3, 6, 12.5, 25, and 50 μg/cm^2^) of each nanomaterial. The day before the experiment, A549 cells were seeded in sterile, flat-bottom 96-well tissue culture plates (Greiner, Pleidelsheim, Germany), in a supplemented RPMI 1640 culture medium (Termo Fisher Scientific), at a cell density of 2 × 105 cells/mL. Cells were incubated at 37 °C and 5% CO_2_. On the next day, the medium was replaced by fresh medium containing the different samples to be analyzed. Each concentration was tested at six wells per plate. The cells were incubated for 48 h; the negative control was the culture medium, and the positive control was a hydrogen peroxide solution (500 μM). After the exposure time, the medium was replaced by propidium iodide (0.3 mM) in culture medium (stock solution in DMSO (1.5 mM), diluted with culture medium 1:5000). Fluorescence was measured (excitation, 485 nm; emission, 590 nm) with a microplate reader (FLUOstar Omega, BMGLabtech, Germany).

### 2.5. Reactive Oxygen Species (ROS) Production

The intracellular ROS was determined using a well-characterized probe, 2′,7′-dichlorofluorescein diacetate (H_2_DCF-DA; Life Technologies, Cramlington, UK) according to a procedure previously described [65], with some adaptations. Briefly, the A549 cell line was seeded at a density of 2 × 10^4^ cells/well in each well of a 96-well plate, in 100 μL of supplemented RPMI 1640 culture medium (Termo Fisher Scientific), and then incubated for 24 h at 37 °C. After that, the cells were pre-incubated for 30 min with 20 μM of H2DCF-DA, in the dark at 37 °C. Then, the probe solution was removed, and fresh medium containing the different nanofibers to be tested was added at six final concentrations of 1.5, 3, 6, 12.5, 25, and 50 μg/cm^2^, in three replicates. A 500 μM hydrogen peroxide solution was used as the positive control for the induction of ROS in cells, and the cell culture medium alone was used as the negative control. The cells were incubated in the presence of the treatments for 1 and 24 h at 37 °C. The DCF levels were determined at an excitation of 485 nm and emission of 520 nm wavelengths using a fluorescence microplate reader (FLUOstar BMGLabtech, Ortenberg, Germany). Data from three independent experiments were reported as mean relative ROS levels expressed as fold-change compared to ROS levels in the respective control cells (fluorescence of exposed cells/fluorescence of unexposed control from the same experimental conditions).

### 2.6. Cytokinesis-Blocked Micronucleus (CBMN) Assay

The CBMN assay was performed according to the OECD 487 guidelines [66] and adapted to overcome the interference of NMs [51]. Briefly, the A549 cells were plated at 2 × 10^5^ cells/well in a 6-well plate and allowed to adhere for 24 h at 37 °C in a humidified atmosphere of 5% CO_2_. After the exposure to 1.5, 3, 6, 12.5, 25, and 50 μg/cm^2^ of each NM for 24 h, cytochalasin B (Sigma) was added to each well at a final concentration of 6 μg/mL, and the cells were incubated for another 24 h period. For each experiment, negative (non-treated cells) and positive (0.05 μg/mL mitomycin C, Sigma) controls were included. At the end of the 48 h treatment, the cells were washed twice with PBS, detached with trypsin-EDTA, and then submitted to a hypotonic shock with a solution of RPMI 1640:dH2O:FBS (37.5:12.5:1). After that, the cell suspension was centrifuged, and the pellet was spread onto microscope slides, which were then dried, fixed in absolute methanol (Sigma), stained with 4% Giemsa (Merck, Darmstadt, Germany), and air-dried at room temperature. The resulting Giemsa-stained slides were coded and blind-scored under a bright field microscope (Axioskop 2 Plus, Zeiss, Germany) for the presence of micronuclei (MN), using the criteria described by Fenech (2007). At least 2000 binucleated cells from two independent cultures were scored per treatment condition, and the frequency of micronucleated binucleated cells per 1000 cells (MNBC/1000 BC) was determined. The proportion of mono-(MC), bi-(BC), or multinucleated cells (MTC) was calculated by scoring 1000 cells per treatment, and the cytokinesis blocked proliferation index (CBPI) was calculated as follows [67]:

(3) $$\mathrm{CBPI}=\frac{\mathrm{MC}+2\mathrm{BC}+3\mathrm{MTC}}{Total\;cells}$$

### 2.7. Cellular Uptake by TEM Imaging of Cells Exposed to CMNM

The A549 cells were seeded onto glass cover slips (1 × 10^5^ cell/mL) at 37 °C in an atmosphere of 5% CO_2_ for 24 h before exposure to each CMNM and MWCNT under study (25μg/cm^2^). After a 24 h exposure, the cells were initially fixed by adding a solution of 2% formaldehyde (EMS #15710) and 2.5% glutaraldehyde (EMS #16220) in 0.1 M phosphate buffer to an equal volume of the culture medium for 15 min (final concentration 1% formaldehyde, 2.5% glutaraldehyde in 0.05 M phosphate buffer). Then, the cells were washed twice with 0.1 M phosphate buffer, fixed in 2% formaldehyde and 2.5% glutaraldehyde in 0.1 M phosphate buffer for 1 h at room temperature, rinsed with buffer and then fixed in 1% Osmium (EMS #19110) in 0.1 M phosphate buffer for another hour, on ice and in the dark. The cells were stained with 1% tannic acid (EMS #21700) for 20 min on ice, followed by 0.5% uranyl acetate (Analar #10288) for 1 h in the dark. The staining step was followed by dehydration with increasing concentrations of ethanol (30–100%). After this, the cells were embedded in EMbed-812 (EMS #14120) epoxy resin, and the blocks were polymerized overnight at 60 °C. The embedded samples were sectioned at 70 nm and stained with uranyl acetate and lead citrate for 5 min each before being analyzed with a Tecnai G2 Spirit BioTWIN Transmission Electron Microscope (TEM) from FEI operating at 120 keV and equipped with an Olympus-SIS Veleta CCD Camera.

### 2.8. Statistical Analysis

At least three independent experiments for cytotoxicity and ROS formation were conducted for each type of nanofiber. Test results for each assay were expressed as the percentage of the unexposed control ± standard deviation (SD). Statistical significance was set at *p* < 0.05. Normality of data was confirmed with Q–Q percentile plots and Kolmogorov–Smirnov tests. The equality of variances was evaluated using Levene’s test. One-way analysis of variance (ANOVA) followed by Dunnett’s multiple comparison tests were carried out for normally distributed results with homogeneous variances. Non-parametric tests, namely the Kruskal–Wallis followed by the Mann–Whitney U tests, were applied to results without normal distribution and/or inhomogeneous variances. The 2-tailed Fisher’s exact test was applied to analyze the results of the frequency of micronucleated cells. All analyses were performed with the SPSS statistical package (version 22, SPSS Inc. Chicago, IL, USA).

## 3. Results

### 3.1. Characterization of Nanocellulose

**Figure 2:**
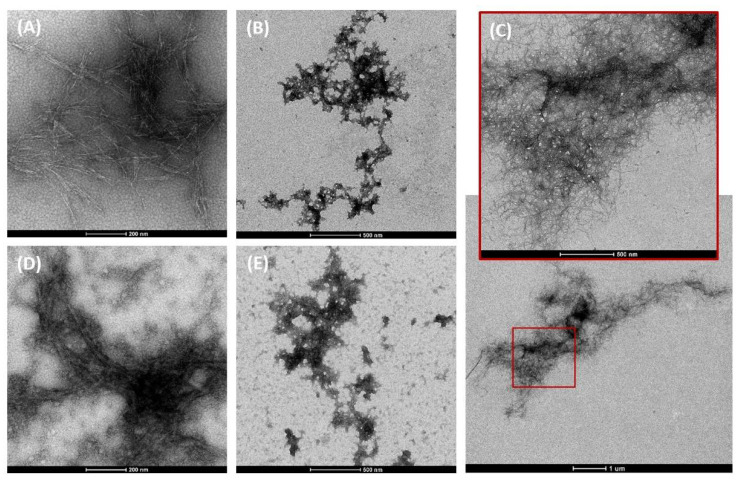
TEM images of the three cellulose micro/nanomaterials under study dispersed in PBS (**A**–**C**) or in complete culture medium (**D**,**E**). (**A**,**D**) CNC; (**B**,**E**) CMF-ENZ; (**C**) CNF-TEMPO.

The properties that are likely to be the most relevant for the cytotoxic and genotoxic effects of the three CMNM under study are presented in Table 2. Fibrillation yield was 100% for CNF-TEMPO, while CMF-ENZ presented only 4.9% of fibrils at the nanoscale. CNF-TEMPO presented a higher content of carboxyl groups, a lower degree of polymerization, and a lower intrinsic viscosity as compared with CMF-ENZ. These parameters were not determined for the CNC. The fibril diameter was estimated by TEM imaging using the software ImageJ, with the CMNM diluted in PBS and in a culture medium. The CNF-TEMPO diluted in PBS presented the lowest diameter (10.7 ± 1.9 nm) and the nanofibrils of CMF-ENZ the highest. For CNF-TEMPO diluted in a culture medium, it was not possible to determine the diameter due to the presence of proteins with the same size and shape that camouflaged the nanofibrils in the TEM images. The presence of these proteins also explains the higher diameter of the CMF-ENZ and CNC samples dispersed in complete culture medium (containing all supplements and FBS) as compared to those dispersed in PBS. The length of the nanofibrils could not be measured by TEM since they are several micrometers long and due to their tangled shape forming aggregates with a high aspect ratio (Figure 2C). 

The zeta potential values obtained for the three CMNM were all negative (Table 2), with the most negative sample corresponding, as expected, to that with the highest amount of carboxyl groups linked to the cellulose chain (CNF-TEMPO). Zeta potential measurements of the nanocellulose dispersions in the culture medium were less negative than in PBS, which is possibly related to the anchorage of proteins to the surface of the nanofibrils that formed the protein corona.

The morphology of the CNC and CMF-ENZ diluted in PBS and in the culture medium and that of the CNF-TEMPO diluted in PBS can be observed in the TEM images of Figure 2. As expected, the three types of CMNM present evident differences in shape, structure, and size. The CNC sample is characterized by short filaments organized into crystal aggregates with a more rigid structure (Figure 2A). A completely different structure is observed for the CMF-ENZ sample, where the nanofibers are shorter in length, closely bundled, and forming branches (Figure 2B). With the TEMPO-mediated oxidation pre-treatment, the resultant CNF-TEMPO present a reduced diameter and higher length and are highly tangled (Figure 2C). The CNFs produced by this process have a higher surface area than the CMFs obtained with the enzymatic pre-treatment. Some differences were also found between the structure of the nanofibers CMF-ENZ and CNC diluted in PBS and in the culture medium. As mentioned before, the medium is rich in proteins that become linked to the nanofibrils’ surface, thus changing their structure and their electrostatic configuration.

The key intrinsic physicochemical properties of the two MWCNT and the secondary properties measured after dispersion in the cell culture medium have been previously described by Louro et al. [52] and Tavares et al. [64]. NM-401 is the thickest and longest MWCNT to have been tested (average diameter and length of 67 and 4048 nm, respectively), displaying an aspect ratio of 53.6 ± 2 and a specific surface area of 140 m^2^/g. It has a straight-wall morphology, i.e., low flexibility, whereas NM-402 is highly bended. The latter presents smaller dimensions (average diameter and length of 11 and 1372 nm, respectively), a higher aspect ratio (107.1 ± 1.9), and specific surface area (226 m^2^/g) [51,52]. After the dilution of batch dispersions in the cell culture medium, a general increase in the size of the agglomerates was noted, followed by the deposition of the largest ones (assessed by DLS measurements and bright field microscopy). This deposition effect was more accentuated for NM-402 than for NM-401 [52].

### 3.2. Cellular Uptake

**Figure 3:**
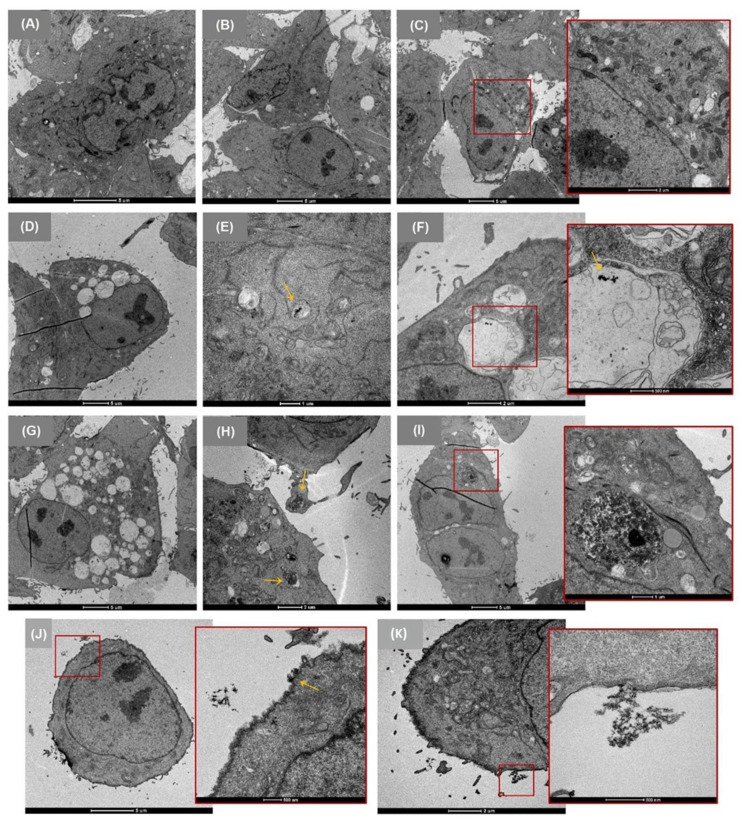
Representative TEM images of A549 cells after 24 h exposure to the CMNM under study (25 μg/cm^2^). (**A**–**C**) Unexposed control cells; (**D**–**F**) CNF-TEMPO exposed cells; (**G**–**I**) CMF-ENZ exposed cells; (**J**,**K**) CNC exposed cells. Yellow arrows indicate the location of the CMNM uptake.

**Figure 4:**
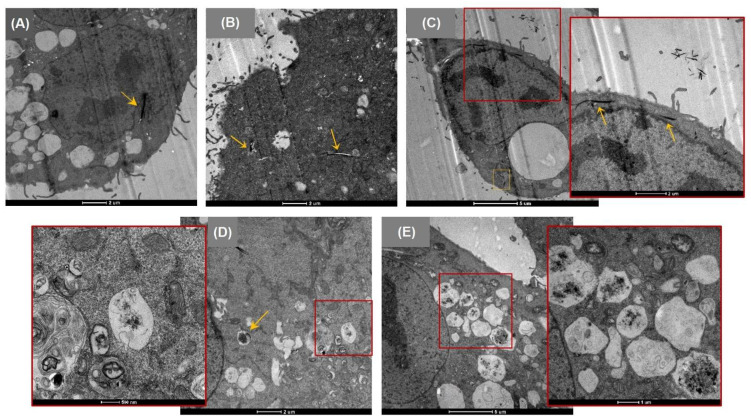
Representative TEM images of A549 cells after 24 h exposure to the MWCNT under study (25 μg/cm^2^). (**A**–**C**) NM-401 exposed cells; (**D**,**E**) NM-402 exposed cells; Yellow arrows indicate the location of the CMNM uptake.

TEM was used to study the interaction of CNF-TEMPO, CMF-ENZ, and CNC (Figure 3), or that of NM-401 and NM-402 (Figure 4) with A549 cells exposed to a concentration of 25 μg/cm^2^ for 24 h, especially to determine their potential internalization and accumulation in cells. In general, morphological differences between the treated cells compared to untreated cells were observed. Control A549 cells (Figure 3A–C) presented the characteristic polygonal morphology with a regular ultrastructure, cluster formation, well-preserved cytoplasm, intact organelles, and numerous well-defined mitochondria [68]. The images revealed that the cells exposed to CNF-TEMPO (Figure 3D–F), CMF-ENZ (Figure 3G–I), NM-401 (Figure 4A–C), and NM-402 (Figure 4D–E) presented phenotypic changes such as a higher number of cytoplasmic or endocytic vacuoles, binucleation (Figure 4I), and surface finger-like protrusions, which may evidence the internalization of these NM through engulfment and endocytosis. The CNF-TEMPO (Figure 3F), CMF-ENZ (Figure 3I), and NM-402 (Figure 4E) were well-identified inside the endosomes, while the NM-401, which is the most rigid nanofiber, appeared to be free in the cytoplasm, indicating a needle-like penetration and almost reaching the nucleus (Figure 4A). From all NM under study, CNC was the only one that did not evidence cell internalization, mostly remaining at the cell’s surface (Figure 3D–E). 

### 3.3. Cytotoxicity

**Figure 5:**
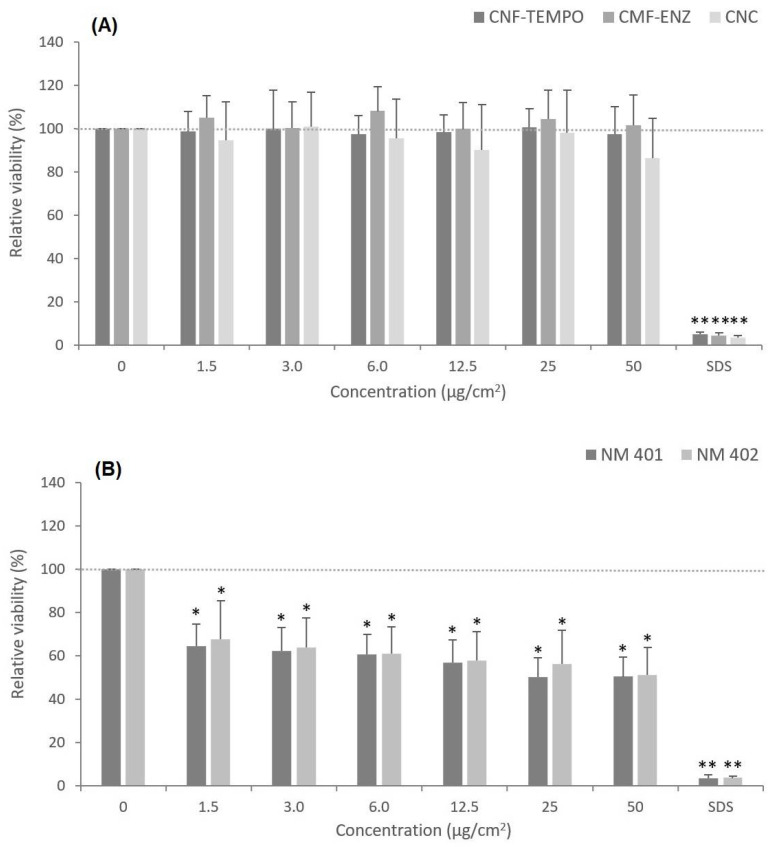
Effects of nanocellulose and MWCNT exposure at different concentrations on the viability of A549 cells assessed by the MTT assay, after 24 h exposure: (**A**) CNF-TEMPO, CMF-ENZ, and CNC; (**B**) NM-401, NM-402. The results are expressed as mean ± SD of three independent experiments, each carried out in triplicate. * *p* < 0.05, ** *p* < 0.01.

The potential cytotoxic effect of CMNM was assessed in comparison with two reference MWCNT (NM-401 and NM-402) by the MTT cell viability assay (Figure 5). The results showed that after a 24 h exposure period, none of the three CMNM, at concentrations ranging from 1.5 μg/cm^2^ to 50 μg/cm^2^, induced a significant cytotoxic effect in A549 cells as compared to the controls (*p* > 0.05). On the contrary, after the same length of exposure and concentrations, both MWCNT induced a significant decrease in A549 cell viability. The positive control, SDS 0.1 mg/mL, induced a statistically significant decrease in the number of living cells in all the experiments.

**Figure 6:**
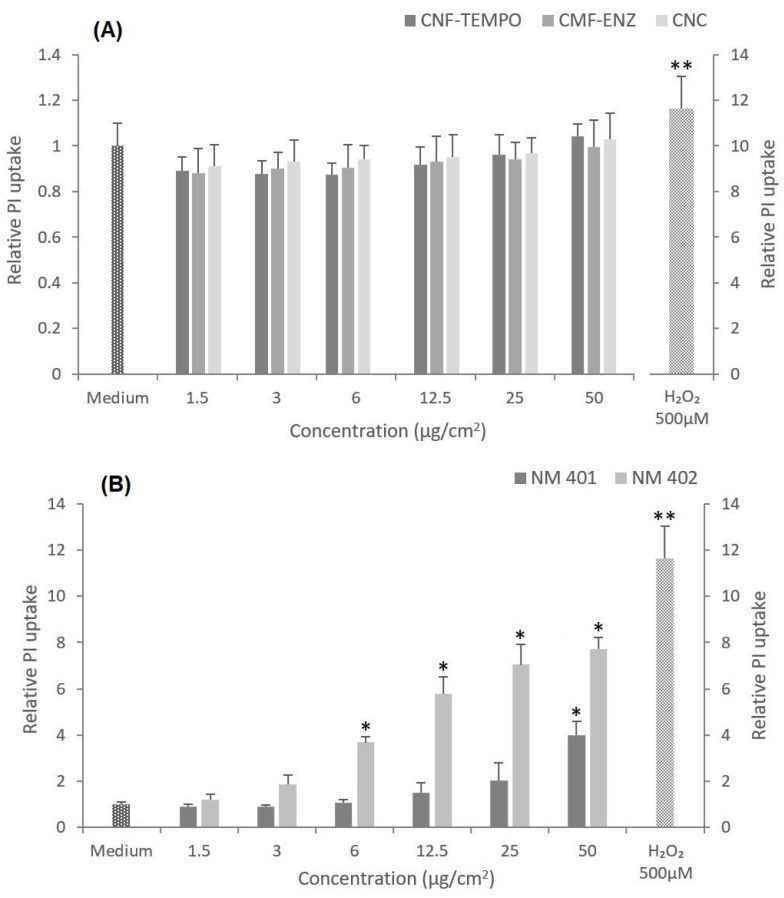
Effects of nanocellulose and MWCNT exposure at different concentrations on the cell viability of A549 cells assessed by the propidium iodide (PI) membrane integrity assay, after 24 h: (**A**) CNF-TEMPO, CMF-ENZ, and CNC; (**B**) NM-401, NM-402. Data are the mean ± SD of three independent experiments. * *p* < 0.05, ** *p* < 0.01.

Furthermore, the CMNM cytotoxicity was complementarily assessed by the propidium iodide (PI) membrane integrity assay, after a 24 h exposure to the same concentration range used for assessing cell metabolic activity (Figure 6). The exposure to all tested CMNM did not result in significant increases in fluorescence values as compared with the control, indicating that PI did not penetrate the cell membrane of cells, i.e., they are alive. On the contrary and as expected, there was a significant dose-dependent increase in the relative PI uptake by the cells after exposure to the two tested MWCNT, in particular to the NM-402.

**Figure 7:**
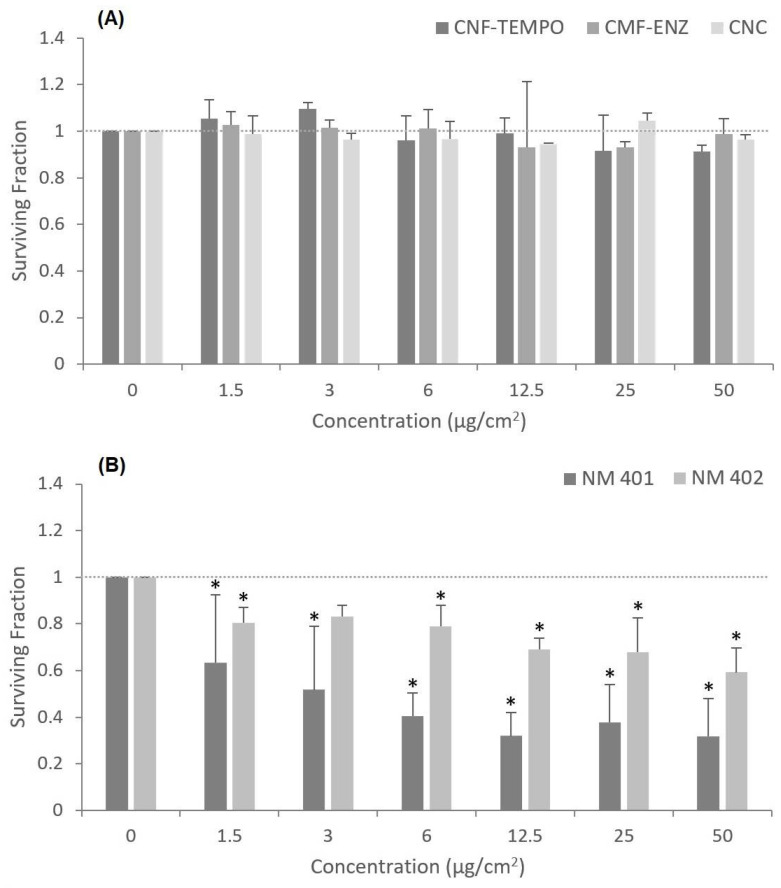
Effects of nanocellulose and MWCNT exposure (eight days) at different concentrations on colony formation of A549 cells: (**A**) CNF-TEMPO, CMF-ENZ, and CNC; (**B**) NM-401, NM-402. The results are expressed as mean ± SD of three independent experiments, each carried out in triplicate. * Denotes a statistically significant difference from the control (*p* < 0.05).

A clonogenic assay was also performed to evaluate the capacity of the three CMNM to prevent the cells’ colony-forming ability after a longer exposure period in comparison with the NM-401 and NM-402. The results (Figure 7) are in accordance with those obtained by the MTT and the PI assays, demonstrating that all tested nanocelluloses were unable to significantly decrease A549 cells’ replication capacity when exposed constantly over eight days. Moreover, a statistically significant and dose-dependent decrease in the number of A549 colonies formed was observed for the two MWCNT (*p* < 0.05), which is more evident for the NM-401. The exposure to the positive control (Mitomycin C, 0.05 μg/mL) resulted in a decrease in the cell’s capacity to form colonies by more than 50% relative to control (*p* = 0.0002).

### 3.4. Oxidative Stress

**Figure 8:**
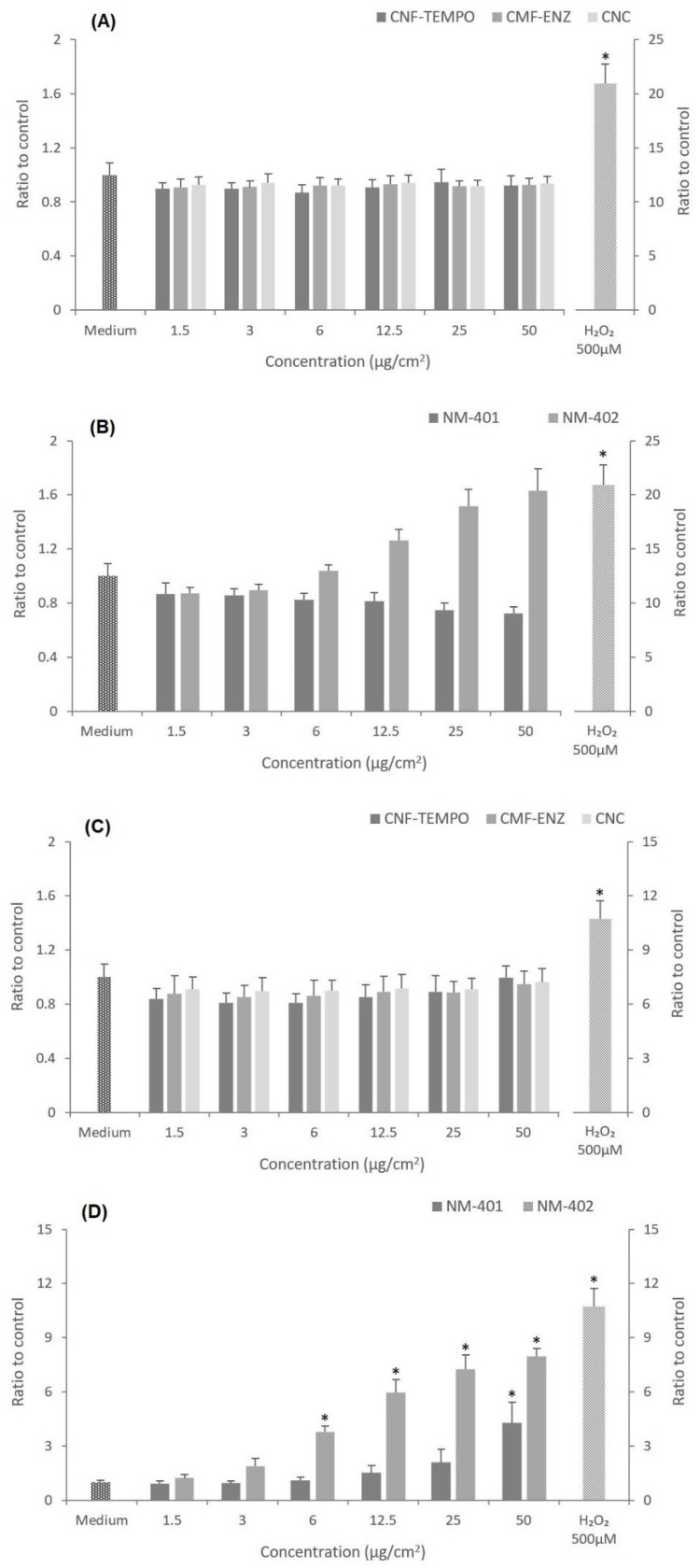
Intracellular reactive oxygen species (ROS) production after 1 h and 24 h exposure of A549 cells to nanocellulose and MWCNT samples under study: (**A**) 1 h exposure to CNF-TEMPO, CMF-ENZ, and CNC; (**B**) 1 h exposure to NM-401 and NM-402; (**C**) 24 h exposure to CNF-TEMPO, CMF-ENZ, and CNC; and (**D**) 24 h exposure to NM-401 and NM-402. The results are expressed as mean ± SD of three independent experiments, each carried out in triplicate. * Denotes a statistically significant difference from the control (*p* < 0.05).

Intracellular reactive oxygen species (ROS) production in A549 cells following exposure to CMNM or to MWCNT for 1 and 24 h was evaluated through the presence of 2′,7′-dichlorofluorescein (DCF) fluorescence (Figure 8). No significant intracellular ROS increase was observed in the A549 cells exposed for 1 h to all tested samples as compared with the negative control (*p* > 0.05). Although treatment with NM-402 tended to raise ROS levels at concentrations of 12.5, 25, and 50 μg/cm^2^ (Figure 8B), the increases did not reach statistical significance over the control level (*p* = 0.886, *p* = 0.332, and *p* = 0.174, respectively). After a 24 h exposure, the three CMNM and NM-401 did not significantly increase intracellular ROS production, while NM-402 induced a significant concentration-dependent increase in ROS. The basal level of intracellular ROS in non-exposed cells (negative control) was low, but on the contrary, the positive control (H_2_O_2_ solution) induced a significant increase in DCF fluorescence as compared with the negative control (*p* = 0.0001).

### 3.5. Genotoxic Effects

**Figure 9:**
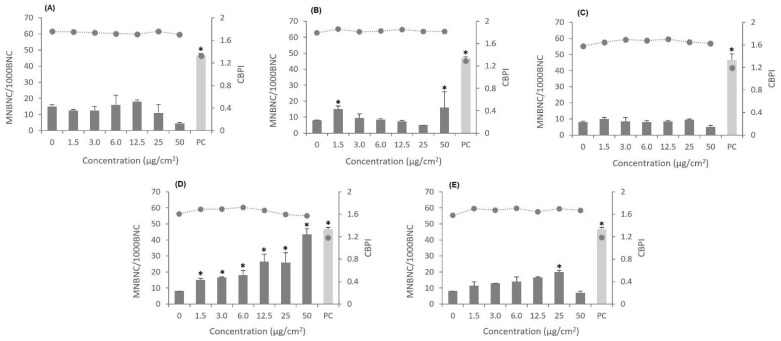
Results of the micronucleus assay after A549 cells’ exposure to the three nanocelluloses: (**A**) CNF-TEMPO, (**B**) CMF-ENZ, and (**C**) CNC, and to the two MWCNT: (**D**) NM-401, (**E**) NM-402 at different concentrations. Columns represent the frequency of micronucleated binucleated cells (MNBC) per 1000 binucleated cells (BNC); the dotted line represents the cytokinesis-blocked proliferation index (CBPI). Mitomycin C, 50μg/mL was used as the positive control (PC). The results are expressed as mean ± SD. * Denotes a statistically significant difference from the control (*p* < 0.05).

The genotoxic effects of the three CMNM under study in comparison with the two MWCNT, NM-401 and NM-402, were assessed by micronucleus assay. The results presented in Figure 9 show that the exposure of A549 cells (1.5 to 50 μg/cm^2^, 48 h) to CNF-TEMPO (for the concentrations of 6 and 12.5 μg/cm^2^, *p* = 0.499 and *p* = 0.898, respectively) and to CNC or to NM-402 did not produce significant alterations in the frequency of micronucleated binucleated cells (MNBC) as compared to the control. There is, however, a significant increase in the MNBNC frequency induced by the CMF-ENZ at the concentrations of 1.5 and 50 μg/cm^2^ (*p* = 0.0014 and *p* = 0.0005, respectively) (Figure 9). In contrast, NM-401, the thickest, longest, and most rigid nanofiber, was able to significantly increase the frequencies of MNBNC for all concentrations tested. The cytokinesis-block proliferation index (CBPI) of the A549 cells was not affected by nanocellulose or MWCNT exposure (Figure 9). The positive control (Mitomycin C, 0.05 μg/mL) induced a statistically significant increase in the frequency of micronucleated cells as compared with the negative control in all experiments performed (*p* = 0.00001). 

An overview of the results obtained for the endpoints analyzed is presented in Table 3.

## 4. Discussion

CMNM have demonstrated a great potential for use in multiple applications in different industrial fields due to their unique properties. Therefore, the wide production and use of CMNM has led to the rapid increase in the number of people exposed to this kind of NM, in environmental and occupational settings or via consumer products. Inhalation is considered to be the main route of human exposure to this kind of NM, and the lungs are the primary target organ for toxicological effects. Despite cellulose often being considered as non-toxic due to its natural origin, the nanoscale dimension and intrinsic properties of these materials at the nanoscale raise concerns about their potential effects on human health. Consequently, it is necessary to conduct nanotoxicological studies in order to evaluate the safety of CMNM and minimize the risks associated with their use before scaling up their production and introducing them into the market. There has been an increasing number of in vitro/in vivo toxicological studies performed during the last few years, although the knowledge on the potential hazards of CMNM to human health is still limited and sometimes based on contradictory findings. It is difficult to compare and make a distinction between the toxicity of different types of CMNM (e.g., fibrils vs. crystals) that is resultant from different sources of raw materials, isolation procedures, processing/manufacturing procedures, drying methods, and type of surface functionalization. These variables impart key physicochemical properties such as size, shape, dimensions, and surface reactivity, which influence the toxicological profile of CMNM. In this study, we evaluated and compared the in vitro toxicity of three different types of CMNM, all derived from industrial BEKP and presenting different morphologies and surface structures, in A549 cells under the same experimental conditions. The in vitro toxic effects of two other fibrous NM, NM-401 and NM-402 from the JRC Repository, were also assessed because of their inherent fiber-like morphology and thus their structural resemblance to the CMNM under study. 

The CNF-TEMPO sample was obtained by a chemical/mechanical procedure, as described above. The chemical step consisted of a TEMPO-mediated oxidation that contributed to the conversion of the C6 primary hydroxyl groups of the glucose units into carboxylic groups at the surface of the nanofibrils. A high amount of carboxylic groups was achieved at 1332 μmol/g, as determined by conductometric titration, which provided high electrostatic repulsion between the nanofibrils. The subsequent mechanical step consisting of high-pressure homogenization led to the separation of the individual fibrils, resulting in a small degree of polymerization and in an aqueous gel-like appearance that was completely transparent. The CMF-ENZ consisting of cellulose microfibrils produced by an enzymatic hydrolysis process and followed by HPH were also characterized. Enzymatic hydrolysis is an environmentally friendly pre-treatment that does not generate toxic residues but induces the breakdown of cellulose polymer into smaller polymer branches, thus favoring the posterior mechanical treatment [69]. The fibrillation yield of the CMF-ENZ sample was low (4.9%) when compared with the nanocellulose obtained with the chemical pre-treatment [58,70]. Moreover, the concentration of carboxyl groups was much lower (143 μmol/g), as expected, since no oxidation treatment was applied, which resulted in a higher degree of polymerization. The third type of CMNM under study was obtained by the acid hydrolysis method with sulfuric acid and consisted in cellulose nanocrystals (CNC). The chemical composition of the CNC was assessed by elemental analysis, whose results allowed for a speculation on the validity of the esterification reaction that occurs within the hydrolysis process. In this process, ester-sulfate groups were covalently linked to the surface of CNC, allowing for the stability of the nanoparticles in the suspension [71]. The wt percentages achieved for C, H, S were 41.65, 6.06, 0.63%, respectively. These values are in line with those observed by the group for samples produced from the same source and under similar hydrolysis conditions [60]. 

Besides the relevance of a good physicochemical characterization of the NMs, the use of well-controlled dispersion methods to prepare homogeneous and stable stock dispersions of insoluble NMs is also central to obtaining reliable and reproducible toxicological responses. In this study, different dispersion methods, all using an aqueous dispersion medium, were used to prepare the stock dispersions of CMNMs or MWCNTs, with the main intention of obtaining the best and most stable stock dispersions, and which were subsequently diluted in the cell culture medium in order to expose the cells. TEM imaging was used to confirm the nanometric scale of the CMNM and to analyze their morphology and structure when dispersed in PBS and in complete cell culture medium, given that the referred properties can influence the biological effects of these NM in the presence of cells. Regarding NM-401 and NM-402 dispersed in 0.05% BSA-water solution, previous DLS analyses had shown a good dispersion after sonication, and a better understanding of the MWCNTs’ characteristics was obtained by TEM analyses; more details can be found in previous publications [51,64]. Among CMNMs, all nanofibers presented nanometric thickness, and the CNF-TEMPO displayed the lowest average diameter (10.7 ± 1.9 nm). An evident change was observed in the morphology of the CNC and CMF-ENZ samples diluted in a protein-rich culture medium, which was probably related to the binding of proteins at their surface, forming the so-called protein corona. This was also verified by an increase in the z-potential results of these two nanofibers to values that were less negative than those obtained with the samples diluted in PBS. The protein adsorption is influenced by the surface chemistry of the nanomaterial that determines the type, conformation, and amount of proteins adsorbed at its surface, and this, in turn, may impact on the agglomeration state of the nanomaterial that may indirectly affect the cellular response [72,73]. Similarly, the temporal evolution and average hydrodynamic size of the dispersed NM-401 and NM-402 diluted in the same protein-rich cell culture medium have been thoroughly studied and published elsewhere [64]. Despite the use of a standardized procedure for these MWCNT dispersions, DLS and TEM analyses revealed the presence of many agglomerates after dilution in the culture medium, possibly due to the formation of a protein corona and the sedimentation of the coarsest particles, as observed for the CMNMs. Therefore, it is likely that both classes of NMs will form a protein corona upon dispersion in the culture medium, irrespectively of the dispersion procedure used to prepare their stock dispersion.

The cellular uptake of the NM under study was investigated using TEM imaging. This electron microscopy technique enables the identification of the localization of NM in the intracellular environment and provides information at the ultrastructural level of the cell system [74]. Through TEM analysis, CNF-TEMPO and CMF-ENZ and both MWCNTs were observed in the cells´ endosomes and cytoplasm, respectively, whereas no evidence of CNC internalization was seen. Intracellular modifications occurred in A549 cells after exposure to two CMNM and to the two MWCNTs under study. The cells treated with CNF-TEMPO, CMF-ENZ, and NM-402 exhibited more cytoplasmic or endocytic vacuoles, suggesting that the uptake mechanism occurred by endocytosis. This process involves the invagination/ruffling of the cell membrane, followed by the formation of intracellular/endocytic vesicles; one common feature is the localization of NM in endocytic vesicles after internalization [75]. Despite the perceived cellular uptake of CNF-TEMPO, suggestive morphological changes were only observed in very few cells (less than 50%), indicating minimal cellular internalization. These results are in agreement with those reported by Li et al. 2021, who also detected a low cellular uptake and consequent low cytotoxicity of longer CNF in liver cells. On the contrary, about 80% of observed cells contained significant amounts of CMF-ENZ and NM-402 inside the cells, mostly seen in endosomes, and NM-401 in cytoplasm, but none were found in the nuclei. Interestingly, no cellular uptake was evidenced for the cellulose nanocrystals. CNC aggregates were observed outside, mostly localized at the boundaries of A549 cells. Contradictory results about CNC internalization have been reported in literature. In a recent study, Kisin et al. 2020 [13] showed by TEM imaging that CNC powder and gel (30 μg/cm^2^) were internalized by BEAS-2B cells after a long exposure period of 4 weeks. Likewise, after a 72 h exposure, the cell uptake of the CNC powder and gel by A549 cells was revealed by a specific type of cellulose staining [45]. However, confocal laser scanning microscopy (CLSM) demonstrated that the negatively charged CNC-FITC was negligibly internalized by human embryonic kidney 293 (HEK 293) cells [76], by C6 rat glioma and NIH3T3 normal fibroblasts [77], and by nine different cell lines (HBMEC, bEnd.3, RAW 264.7, MCF-10A, MDA-MB-231, MDA-MB-468, KB, PC-3, and C6) [78]. There is an unfavorable interaction between negatively charged NM and the negatively charged cell membrane, which explains an inferior rate of endocytosis [79]. However, there is evidence of cell uptake by negatively charged NM [79,80,81,82] as well as by the CNF-TEMPO and CMF-ENZ in this study. Another important factor that may justify the absence of cell internalization in the characterized CNC is the aforementioned formation of a protein corona. The composition of a protein corona is an important determinant of the fate and cellular internalization of NM, and this can contribute to the reduction of the cellular uptake of functionalized NM by shielding the ligands from binding to their receptors [75]. Furthermore, the cellular uptake depends on several factors, including NM properties such as composition, size, shape, stiffness, and surface chemistry as well as their concentration, exposure duration, and the cell type itself [75,79,81]. The mechanism of NM-401 cellular uptake appears to be different from endocytosis. In this case, the internalization may occur through an energy-independent needle-like penetration, as already described for MWCNT [83,84]. Rubio et al. 2016 [53] demonstrated by TEM imaging the cell uptake of NM-401 (dose-range of 0.12 to 12 mg/cm^2^) in Chinese hamster lung (V79) fibroblasts after a 24 h exposure period. The structure of MWCNT strongly affected their interactions with the cell membrane, implying a possibly shape-dependent uptake process [79]. It has been demonstrated that rigid MWCNT behave similarly to nano-needles that can pierce or penetrate through the cell membrane and into the cytoplasm [85,86]. 

The cell viability, following exposure to the three CMNM under study and in comparison with the two selected MWCNT, was analyzed in A549 cells through three assays covering different endpoints. The MTT assay evaluated the alteration of the cells’ metabolic activity, the clonogenic assay assessed the cells’ proliferative ability, and the PI assay considered the loss of membrane integrity. All assays showed agreement in the results, which indicated no toxicity after exposure to the CMNM. On the contrary, the MWCNTs NM-401 and NM-402 revealed a dose-dependent cytotoxic effect following the same exposure conditions (24 h or eight days, 1.5–50 μg/cm^2^). Despite our study implying that CMNMs are nontoxic to lung epithelial cells, there are reports in the literature that show some divergent findings. Menas et al. 2017 [45] reported a significant decrease in cell viability after a 72 h exposure to a CNF powder and a CNF gel, both at 1.5, 15, and 45 μg/cm^2^, which was significantly higher as compared to a 24 h exposure. However, in agreement with our results, it was shown that the tested CNC samples were not cytotoxic in A549 cells [45]. In another study, Ventura et al. 2018 [16] reported the capacity of one CNF produced by the same TEMPO-mediated oxidation process to induce alveolar cell death in a dose-dependent way, and more significantly at 25 μg/cm^2^ following a 48 h or eight-day exposure. However, none of the CNF concentrations tested induced a significant cytotoxic effect in A549 cells after a 24 h exposure [16]. In addition, a preliminary study with A549 cells exposed to a similar concentration range of the CMF-ENZ and CNF-TEMPO used in this study for 24 h, 48 h, and 72 h showed no significant variation in cell viability, irrespectively of the duration of exposure [87]. Likewise, Yanamala et al. 2016 [36] verified no significant decrease in cell viability in A549 cells upon exposure to CNC, CNF, L-CNC (Lignin-CNC), and L-CNF (Lignin-CNF) at 5–300 μg/mL after 24 h and 72 h, while a dose-dependent cytotoxic effect was observed in THP-1 cells. Other in vitro studies have addressed the cytotoxic potential of CMNM in different cell lines, and the majority reported nontoxic effects under conditions of exposure time and concentrations approximate to those applied in this study [76,78,88,89,90,91,92,93,94]. With respect to the long-term cytotoxic effects of the two MWCNT assessed by the clonogenic assay after eight days of exposure, it was observed that the longest, thickest, and most rigid NM-401 (diameter, 67 ± 24 nm; length, 4048 ± 2371 nm) induced more evident effects than the shortest and highly entangled NM-402 (diameter, 11 ± 3 nm; length, 1372 ± 836 nm). However, after a short-term (24 h) exposure of the A459 cells, the cytotoxic effects analyzed by the PI membrane integrity assay were more evident with the NM-402 that was uptaken by endocytosis, as observed by TEM imaging. This nanofiber induced toxicity in the A549 cells, including the loss of metabolic activity, the incapacity to divide, and especially a concentration-dependent alteration of membrane integrity. On the other hand, NM-401 possibly penetrates into cells by piercing the cell membrane, thus triggering cell death. These observations suggest that under the analyzed conditions, important physicochemical aspects such as the rigidity and agglomeration state or the combination of these properties may influence the cytotoxicity of these MWCNT. TEM and DLS analyses have previously shown that although NM-401 and NM-402 dispersions had been successfully achieved, many agglomerates could still be seen. The increasing size of those agglomerates observed with increasing concentrations might explain the lack of a clear concentration–response relationship for the cell viability decrease assessed by the colorimetric MTT assay [64]. Another possibility would be the likely interference of the MWCNTs with the spectrophotometric measurements used in the MTT assay, as has been reported in the literature; for example, this is the case for titanium dioxide NMs [95] but, to our knowledge, not for MWCNT. The results obtained agree with those reported by Louro et al., 2016 [51], showing a decrease in A549 lung epithelial cell proliferation, after an eight-day exposure to concentrations ranging from 16 to 128 μg/cm^2^ with NM-401, and in concentrations ranging from 60 to 128 μg/cm^2^ with NM-402. They proposed that larger or more agglomerated MWCNT were associated with higher cytotoxicity, which in turn may be influenced by different protein coronas in protein-rich biological media. Thus, coated MWCNT have a higher tendency to agglomerate and induce toxic effects as compared to uncoated MWCNT [51,96]. Considering the fiber-like morphology of CMNM, these findings may also apply to this kind of NM. Similar results were reported by Di Cristo et al., 2019 [97], who verified pronounced cytotoxic effects after the 72 h exposure of two distinct macrophage lines (Raw264.7 cells and MH-S macrophages) to NM-401, and contrary results with NM-402 [97]. Other studies, however, reported no cytotoxic effects in A549 cells following exposure to NM-401 using cell counting [53,98] or to NM-402 using the colorimetric WST-1 assay and a concentration range similar to that tested in this study [99], evidencing inconsistent results for the cytotoxic potential of these benchmark MWCNTs. 

In the present work, the oxidative potential of CNF-TEMPO, CMF-ENZ, and CNC was assessed and compared with that of NM-401 and NM-402 using the fluorescent marker H2DCF-DA. No significant 2′,7′-dichlorofluorescein (DCF) fluorescence increase was observed after A549 cell treatment with the three CMNM for 1 h and 24 h as compared with the negative control. Under the same experimental conditions, a significant release of intracellular ROS in A549 cells was triggered by the NM-402 and NM-401 (50 μg/cm^2^) only after a 24 h exposure. Similar results were obtained by Jackson et al. 2014 at concentrations between 1.4 and 200 mg/mL, after a 3 h incubation period [100]. The evident cellular uptake of NM-401, and especially of NM-402, could have contributed to the ROS formation, which in turn may have promoted the cytotoxicity that was associated with the MWCNT treatment. A bioaccumulation effect can also be estimated since the production of ROS increased over the exposure time. An excessive increase in ROS can promote oxidative damage due to a disruption of redox homeostasis induced by the deterioration of the ROS-scavenging capacity or by an abnormal elevation of ROS production [13]. The exposure time and the surface modification of CMNM can be important factors to consider for the formation of ROS, which are intimately related with frustrated phagocytosis, particularly for nanofibers with low flexibility [72]. Kisin et al., 2020 [13] reported that the exposure of BEAS-2B cells to a CNC powder and CNC gel (30 μg/cm^2^) for 72 h induced the generation of intracellular ROS. Using the same cell line, Aimonen et al., 2021 [34] verified the formation of intracellular ROS triggered by unmodified CNF (U-CNF) after 24 h, and by carboxymethylated CNF (C-CNF) after 3 h, 6 h, and 24 h of exposure, in concentrations ranging from 2.4 to 312.5 μg/cm^2^. However, the hydroxypropyltrimethylammonium CNF (H-CNF), phosphorylated CNF (P-CNF), or the sulfoethylated CNF (S-CNF) did not induce the production of intracellular ROS at any of these exposure times. In another study, Lopes et al., 2017 [72] showed that the treatment of THP-1 macrophages with U-CNF, C-CNF as well as with H-CNF (in increasing doses of 50–500 μg/mL) resulted in no significant ROS increase after a 2 h exposure. Despite the occurrence of cellular internalization of CMF-ENZ and CNF-TEMPO in a more discreet way, these two CMNM did not present cytotoxic effects nor ROS production, contrary to what was observed with the NM-402, which also has a fibrillar and entangled morphology and was apparently uptaken by a similar endocytic mechanism. This may be related with the toxicity of impurities, especially transition metals (Fe, Ni, Al, S, and Cl) introduced during the preparation and purification of CNT that could be released and may induce toxicity in living organisms, as demonstrated for Ni [100,101]. The effect of longer or repeated exposures will give further insights into CMNMs’ in vitro toxicity, particularly considering that these are biopersistent materials and thereby with the potential to accumulate in the body.

The genotoxic effects of the CMNM and MWCNT under study were investigated by the cytokinesis-blocked micronucleus assay, which assesses chromosome breaks and chromosome loss in binucleated cells, i.e., cells in the first post-mitotic interphase after exposure [41,102]. Among the currently available genotoxicity tests, the micronucleus assay has been widely used due to its reliability in assessing not only chromosomal breaks or the disruption of the mitotic apparatus, but also other events such as DNA amplification (assessed by scoring nuclear buds) and chromosome rearrangements (nucleoplasmic bridges), which can be considered hallmarks of genotoxicity and predictive of carcinogenesis [103,104]. However, the sensitivity of this in vitro assay is limited by the variability associated with some factors such as the stage of the cell cycle, the type and repair capacities of target cells, and the time elapsed between exposure and analysis [16,105]. 

The present results showed that after the 48 h exposure of A549 cells, there was no significant increase in the frequency of micronucleated binucleated cells for CNF-TEMPO and CNC, and consequently, no genotoxicity was observed. The lack of clastogenic or aneugenic effects in alveolar cells upon CNF-TEMPO or CNC exposure may be associated with the low or inexistent cellular uptake observed, respectively. These findings increase the weight of evidence in favor of the biocompatibility of these CMNM. There was, however, a significant genotoxic effect detected at the lowest and the highest CMF-ENZ concentrations tested. In theory, this CMNM contains higher amounts of lignin, ash, and hemicellulose, resultant from the enzymatic hydrolysis, when compared with the CNF-TEMPO, which is obtained from a substrate enriched in cellulose and more prone to fibrillation. There are studies demonstrating the in vitro cytotoxic effects of industrial kraft lignin on mouse hepatoma MH-22A, melanoma B16 (tumor cells), and Chinese hamster ovary (CHO, non-cancerous) cells [106] as well as on the kidney cell line (NRK-52E) [107], but no reports exist on its genotoxicity. However, the chemical composition of CMF-ENZ resultant from its production process, together with the fact that it suffers a higher uptake by cells, may justify the obtained genotoxic effects. The NM-402 was uptaken by cells and induced cytotoxicity that was possibly mediated by ROS generation, but a significant genotoxicity was observed only at a fairly high concentration, which may be related to its entangled morphology. In contrast, the NM-401 was able to significantly increase the frequency of MNBNC, which may be related with the aforementioned release of transition metal impurities [100] and with the interference of CNT with chromatin or cytoskeletal filaments during mitosis, as observed for the similarly rigid MWCNT-7 [32]. The CBPI of exposed A549 cells did not show differences as compared with non-exposed cells, suggesting that the tested CMNMs and MWCNT do not affect cells progression through the cell cycle. The lack of in vitro genotoxic effects elicited by another lung cell line’s (BEAS-2B cells) exposure to different CMNM was previously reported in three studies [34,41,107]. None of the assessed CNF (nonfunctionalized, enzymatically pretreated, carboxylated, H-CNF, P-CNF, C-CNF, and S-CNF) were able to induce DNA or chromosomal damage after treating the cells with the nanofibrils in concentrations ranging from 2.4 to 312.5 μg/cm^2^ for 24 and 48 h [34,107]. In addition, neither CNC nor microcrystalline cellulose (MCC) samples increased the frequency of micronucleated cells in mononucleate or binucleate BEAS-2B cells after 48 h of exposure to concentrations ranging from 0.5 to 20 μg/cm^2^ [41]. Nevertheless, using the same cell line, Kisin et al., 2020 [13] reported a DNA strand break increase, detected by the OxiSelect™ Comet assay, after a 72 h exposure to CNC gel and CNC powder samples (30 μg/cm^2^). Moreover, other types of CMNM tested in different cell lines have given conflicting results. De Lima et al. 2012 [49] investigated the genotoxic effects of cellulose nanofibers originating from different cellulose sources (white cotton, brown cotton, ruby cotton, green cotton, and curaua) through the comet assay in human lymphocytes from human peripheral blood and in 3T3 mouse fibroblasts after a 1 h exposure, using nanofiber concentrations of 0.1%. CNF produced from brown cotton and curaua fibers caused DNA damage, while CNF produced from white, ruby, and green cotton proved to be not genotoxic [49]. Using another CNF sample obtained with the same TEMPO-mediated oxidation pre-treatment as that used in the present study, Ventura et al., 2018 [16] reported a low but significant level of DNA damage in A549 cells, in a co-culture with THP-1 cells at 25 μg/cm^2^, and a slight induction of oxidative DNA lesions at 1.5 and 12.5 μg/cm^2^. These authors also found that the two lowest tested CNF concentrations (1.5 and 12.5 μg/cm^2^) were able to significantly increase the frequency of chromosome numerical or structural anomalies in A549 cells through the in vitro micronucleus assay; on the other hand, for the highest concentrations, these effects were not observed [16]. 

Genotoxicity assays evaluating endpoints other than chromosomal alterations such as DNA strand breaks (e.g., the comet assay) or mutations (e.g., the HPRT test) are still needed in order to obtain a more comprehensive assessment of CMNM genotoxicity. These assays will provide additional data on the possible effects of CMNM on the DNA through different mechanisms of action.

## 5. Conclusions

In summary, our findings show that CNF-TEMPO and CNC, derived from the same cellulose source but differing in the preparation process and physicochemical properties, do not induce cytotoxic and genotoxic effects at concentrations up to 50 μg/cm^2^, as assessed by the in vitro micronucleus assay in human lung adenocarcinoma epithelial cells. The CMF-ENZ tested under the same conditions did not evidence in vitro cytotoxic effects, but it was able to induce genetic damage at the lowest and highest concentrations tested (1.5 and 50 μg/cm^2^). In contrast, the MWCNT tubes tested for comparison were cytotoxic and able to induce strong (NM-401) to mild (NM-402) chromosomal damage levels. TEM analysis revealed that all fibrous-like NM were internalized by cells, whereas CNC remained in cell boundaries with no signs of internalization. NM-402 was the only NM tested that was able to induce ROS formation, although it failed to induce micronuclei formation. On the other hand, CMF-ENZ and NM-401 significantly raised the level of chromosomal damage in the treated cells over the controls but were unable to generate ROS. Thus, it is unlikely that the genotoxicity observed for these NMs is mediated by oxidative DNA damage, thus favoring a direct genotoxic effect. The present results increase the weight of evidence towards CNF and CNC biocompatibility and suggest that although CNF/CMF display similarities with MWCNTs, e.g., with their biopersistence and high aspect ratio, they do not elicit an analogous toxicological response in human cells. Complementary data from other endpoints, such as DNA damage and mutation induction, and the exploration of key cellular and molecular events are needed to allow for a more comprehensive safety assessment of these nanocelluloses and a confirmation of their apparent biocompatibility or drive modifications for the production and marketing of safer micro/nanocelluloses.

## Figures and Tables

**Table 1:** Properties of MWCNT under study.

Multi-Walled Carbon Nanotubes	Thickness ± SD (nm)	Geodesic Length ± SD (nm)	Aspect Ratio ± SD	Specific Surface Area (m^2^/g)
NM-401	67 ± 24 ^a^	4048 ± 2371 ^a^	-	140.46 ^a^
	62.8 ± 1.4 ^b^	3366.4 ± 1.9 ^b^	53.6 ± 2.0 ^b^	-
NM-402	11 ± 3 ^a^	1372 ± 836 ^a^		226.4 ^a^
	10.7 ± 1.3 ^b^	1141.3 ± 2.0 ^b^	107.1 ± 1.9 ^b^	-

^a^: Data from Rasmussen et al. 2014 [50], ^b^: Data from Tavares et al. 2014 [64].

**Table 2:** Properties of the cellulose micro/nanomaterials under study.

Micro/Nanocellulose Sample	Fibrillation Yield (%)	C_COOH_ (μmol/g)	DP	[η] (mL/g)	Fibril Diameter ^1^ (nm)		Zeta-Potential (mV)	
					PBS	CM	PBS	CM
CNF-TEMPO	100	1332	309	130	10.7 ± 1.9	-	−24.6 ± 1.0	−19.7 ± 1.5
CMF-ENZ	4.9	143	1591	618	29.7 ± 7.3	85.2 ± 41.2	−11.6 ± 1.0	−9.4 ± 0.6
CNC	44	-	-	-	19.7 ± 6.1	36.0 ± 9.0	−17.3 ± 0.8	−13.9 ± 0.3

^1^ C_COOH_: Carboxyl group content; DP: Degree of polymerization; [η]: Intrinsic viscosity; CM: RPMI cell culture medium; 1: Estimated by TEM imaging and expressed as Mean ± SD of 10 measurements per image.

**Table 3:** Summary of the results of the cytotoxicity, reactive oxygen species (ROS) formation, and genotoxicity endpoints analyzed.

Assay/Endpoint	CNF-TEMPO	CMF-ENZ	CNC	NM-401	NM-402
MTT	-	-	-	+	+
PI	-	-	-	++	++
Clonogenic	-	-	-	++	+
ROS, 1 h	-	-	-	-	++
ROS, 24 h	-	-	-	-	++
MN	-	+	-	++	(+)
CBPI	-	-	-	-	-

-: negative; (+): equivocal, positive at a single concentration; +: positive, no concentration dependency; ++: positive, with concentration dependency.
